# The importance of individualised care, good communication and trust for reducing nasogastric tube feeding under physical restraint: qualitative multi-informant study

**DOI:** 10.1192/bjo.2024.28

**Published:** 2024-04-17

**Authors:** Sarah J. Fuller, Jacinta Tan, Dasha Nicholls

**Affiliations:** Division of Psychiatry, Imperial College London, UK; Northamptonshire Healthcare NHS Foundation Trust, Northampton, UK; Retired Consultant Child and Adolescent Psychiatrist, Swansea, UK

**Keywords:** Nasogastric feeding, physical restraint, compulsory treatment, restrictive practices, eating disorders

## Abstract

**Background:**

Nasogastric tube (NGT) feeding against a patient's consent is an intervention that clinicians working in specialist mental health in-patient units may need to implement from time to time. Little research has explored clinician, patient and carer perspectives on good practice.

**Aims:**

To use qualitative data from people with lived experience (PWLE), parents/carers and clinicians, to identify components of best practice when this intervention is required.

**Method:**

PWLE and parents/carers were recruited via BEAT UK's eating disorder charity. Clinicians were recruited via a post on The British Eating Disorders Society's research page. Semi-structured interviews were administered, transcribed and thematically analysed.

**Results:**

Thirty-six interviews took place and overlapping themes were identified. Participants spoke in relation to three themes: first, the significance of individualised care; second, the importance of communication; third, the impact of staff relationships. Sub-themes were identified and explored.

**Conclusions:**

Good care evolved around positive staff relationships and individualised care planning rather than standard processes. The centrality of trust as an important mediator of outcome was identified, and this should be acknowledged in any service that delivers this intervention.

Nasogastric tube (NGT) feeding under physical restraint is a highly restrictive intervention that can be used to save someone's life. This predominantly occurs in mental health in-patient settings; however, not all mental health in-patient units are able to facilitate this intervention.^1^ The patient population in which this intervention is most likely to be used is in people with restrictive eating disorders such as anorexia nervosa where their physical health is so compromised by insufficient nutritional intake that life is at risk.^2,3^ At the same time, the ethical and legal principles of patient autonomy, liberty and human rights mean that mental capacity and mental health legislation are clear that even when such coercive and restrictive practices are legally permissible and in a patient's best interests, they should only be used in extremis where other less aversive and intrusive options are not feasible or have failed, and even then employed as little and as briefly as possible. This study is one of a series of studies exploring the prevalence and impact of NGT feeding under restraint during in-patient mental health treatment in England. The aim is to address the evidence gap in NGT under restraint for eating disorders, to develop prevention strategies and best practice guidance regarding this restrictive intervention.

There is surprisingly little research to date on which to base clinical guidance. A qualitative study interviewed eight nursing assistants, who reported that being involved in this clinical intervention was highly distressing.^4^ Another study used a questionnaire to identify patients’ and their parents’ views on NGT feeding, where 29% had consented to this intervention and 63% reported they physically resisted.^5^ In this paper, Neiderman and colleagues made nine suggestions for good practice which focused on the discussions prior to the intervention being needed, good communication, staff training and the need for constant reassessment of the need for the intervention. Our previous work examined the decision-making process of clinicians when this intervention is needed ^6^ and highlighted that patients have been diagnosed with post-traumatic stress disorder (PTSD) as a result of this intervention.^7^ However, there is some practical guidance on how to amend traditional dietetic practice to ensure that when this intervention is needed, it is delivered as quickly and safely as possible,^8,9^ and on clinical practice when this intervention is required on paediatric wards.^10,11^ To further complicate a complex issue, there is some suggestion that coexisting conditions such as autism and emerging personality issues may increase duration of NGT feeding under restraint, with greater difficulty in re-establishing normal eating.^12^ There is variation across eating disorder in-patient units in the UK regarding policies of when to initiate NGT under restraint, and how and when to discontinue it, with some units not delivering this intervention at all whereas others have patients receiving the intervention for over a year.^3^ The aim of this paper was to explore – narratives from people with lived experience (PWLE), their parents/carers and the staff supporting them – best practice for NGT feeding under restraint and to identify what reduces the need for this intervention.

## Method

### Design

This qualitative research is part of a wider project about NGT feeding under restraint in mental health settings. The methodology was co-created with a project steering group, consisting of people with lived experience (PWLE), parents/carer representatives, clinicians and academics. The PWLE and parents/carer representatives suggested initial questions for the topic guides, with wider discussion within the group across two meetings, which enabled reflection and consensus across all members. The group agreed the interviews should end with questions focusing on improving practice to provide research participants with the opportunity to use their experiences for good.

The project steering group advised that PWLE interviews should be individually conducted to maintain a sense of a safe space for any experiences and feelings, whereas for parents/carers, group interviews were preferable to encourage peer support. Finally, for the staff interviews, the steering group recommended interviewing participants from both adult and children and young people's units, across the in-patient mental health multidisciplinary teams (MDTs) that facilitated this NGT feeding under restraint.

### Inclusion and exclusion criteria

Inclusion criteria were: PWLE who were over the age of 16, were at least 1-year post discharge from mental health in-patient care, had received NGT feeding under restraint during their treatment in an in-patient mental health unit within England, were not involved in litigation regarding their treatment, and self-certified as well enough to participate. Parents/carers had all had their loved one receive this intervention in a mental health setting at any age in England. Those who had experienced the intervention solely in paediatric/medical hospitals wards were excluded. Clinicians had worked in an in-patient mental health setting in England where NGT feeding was carried out and had to be part of the multidisciplinary team (MDT) where clinical discussions about this procedure were held.

Exclusion criteria were those who did not meet the above criteria.

### Procedure

Recruitment of PWLE and parents/carers was via online advertising by BEAT, the national eating disorders charity. Clinicians were recruited by advertising on the British Eating Disorders Society (BrEDS) network, which reaches over 2000 clinicians working in the eating disorders field. Potential participants who fulfilled inclusion criteria were sent the participant information sheet and consent form to sign. Once signed consent forms were received, an interview via Microsoft Teams was arranged by the researcher.

The steering group co-produced semi-structured topic guides for the interviews which were conducted via remote meetings, recorded and transcribed using integrated Microsoft Teams software. Prior to each interview, participants were asked to re-confirm their consent verbally and invited to ask any questions regarding their participation. The interview schedule is provided in the Supplementary information available at https://doi.org/10.1192/bjo.2024.28.

### Ethics

The authors assert that all procedures contributing to this work comply with the ethical standards of the relevant national and institutional committees on human experimentation and with the Helsinki Declaration of 1975, as revised in 2008. Ethical approval was granted via Imperial College London's Research Ethics Committee – reference number 21IC7157.

### Data analysis

This research project had a qualitative design, using thematic analysis based on the principles outlined by Braun and Clarke.^13^ Six phases were used to explore patterns and identify themes: (a) initial familiarisation, which was achieved by reading all transcripts multiple times; (b) development of coding frame by a manual line-by-line exploration of the data; (c) validation of coding frame, with corroboration by JT and DN using specific examples; (d) coding of transcripts; (e) triangulation between different types of participants; and (f) validation within research team (all authors) and within the research steering group.

## Results

### Participants

There were 36 participants across people with lived experience (PWLE) (*n* = 7), parents or carers (*n* = 13) and clinicians (*n* = 16).

### People with lived experience

Seven female participants were recruited, with ages ranging from 19 to 54 years old. All had been diagnosed with anorexia nervosa; the shortest reported duration of illness was three years, the longest was over three decades. The number of admissions ranged from 1 to 13, the shortest admission reported as 8 months and the longest was 5 years. For some participants they were recalling experiences that were recent (14 months ago), whereas one participant reported being fed under restraint for the first time three decades ago. They recounted psychiatric admissions across both National Health Service (NHS) and independent sector in-patient units. The adult admissions were predominantly to specialist eating disorder units (SEDUs), whereas the child and adolescent mental health (CAMH) admissions were in a variety of settings including general adolescent units (GAUs), psychiatric intensive care units (PICUs), low secure units (LSUs) and CAMH SEDUs.

### Parents/carers

A total of 13 parents (ten mothers, one stepmother and two fathers) took part across three group interviews. All were parents/carers of daughters who were aged between 12 and 27 years old at the time of receiving the intervention. One daughter had received this intervention only twice, whereas another had a 7-year history of back-to-back in-patient admissions during which this intervention was repeatedly used over many months. These parents’ daughters represented admissions in CAMH GAUs, PICUs and SEDUs and in adult SEDUs only.

### Staff

Sixteen staff members were interviewed, five male and eleven female. The shortest recorded clinical experience working with eating disorder patients in mental health settings was 10 months and the longest was 17 years. Staff were from seven professional backgrounds (psychiatry, psychology, dietetics, occupational therapy, nursing, healthcare assistants and peer support workers). These participants had experience in working in services that spanned NHS and independent sector units, in CAMH GAUs, SEDUs, LSUs, MSUs, PICUs and adult SEDUs, and LSUs.

### Thematic analysis

Three major themes were generated, with significant overlap across the participant groups, and the results are presented accordingly. The first major theme was the significance of individualised care, the second the importance of communication, and the third the impact of staff relationships. Major themes and associated subthemes are represented in Table 1.

**Table 1:** Summary of all the themes identified across participant groups

Major theme	Subtheme
Individualised care	Getting to know the patient and providing care according to individual need and circumstances
Communication	Opportunity to talk and for patients and parents to be heard
	Reminding the patient of why they need this intervention
Importance of staff relationships with patients	Kindness / compassion Trust

### Individualised care

Participants were asked: ‘What would good care look like?’ There were several areas where shared views were identified across all participant groups. PWLE spoke about more individualised care in contrast with ‘one-size-fits-all’ treatment models or protocols. Furthermore, they felt this would encourage less resistance to treatment, with the acknowledgement that goals need to be shared between the team and the individual. Provision of individualised care particularly in negotiating and agreeing plans concerning when and how NGT feeding under restraint could be prevented, was employed when needed and for however long was felt to be necessary, even essential. One parent felt that her daughter being treated without individualisation within a set protocol resulted in the harms of being traumatised and slowing recovery.

‘If you were to work with patients on their own kind of individual plans … I think that could have prevented many cases [of feeding under restraint] that I saw. Understand that your goals and the professional's goals are often different … Especially in the context of, like, “Everyone has to be on the same kind of pathway and same treatment trajectory”. Because I think, that's where a lot of the need to force feed people comes from, when the two sides don't agree’. (Participant 15, PWLE)

‘It seemed like the rationale for this [NGT feeding under restraint] was they haven't complied with eating the hospital food, nor the supplements so the next stage was automatically NGT feeding – and if this was by restraint so be it. It was just the hospital policy, it was not person-centred care at all’. (Participant 24, staff)

‘My daughter is having trauma work now, purely because she wasn't offered individualised care. They put her on a programme that was not achievable and then when she couldn't do it, the restrictions got more and more, she was fed under restraint. I truly believe if they had worked with her and not put her on this programme, she would have recovered months sooner’. (Participant 1, parent/carer)

‘We use an individualised approach and that can be hard to hold boundaries on the ward when one kid gets one thing, and another doesn't but when you explain it they understand. So many kids get transferred to us, stuck, because they have been backed into a corner [from an inflexible treatment programme] and never had any adjustments to meet their needs'. ( Participant 21, staff)

### Communication

Both PWLE and parent/carers spoke about the need for patients to have a safe space to talk about their experience, as they acknowledged that NGT feeding under restraint is a difficult experience for patients.

‘When she was being [NGT] fed she had nobody to talk to … she was told “You are too underweight for therapy” and the reality is, yes she would have used those sessions to voice her distress but it would have been very symbolic – we are here for you and listening to you’. (Participant 5, parent/carer)

‘If I was key working (*) someone who needed restraint feeds, I would always ask them on a daily basis “Do we need to do this today, is there anything that would make it easier today?” and I would always reflect back on the last few feeds and say “I can see just how hard this is for you”, and give them time to talk it over with me’. (Participant 27, staff)

Indeed, PWLE reported valuing conversations with staff that spoke to them as individuals and not just a person with an illness, especially when there was a clear focus on why they were needing to be NGT-fed under restraint each day and how that related to what their specific recovery goals were.

‘There was one carer [clinician] who just got her and spoke to her as a human. She was very maternal, and that was so important. Too many conversations were not to her but to her illness and you are never going to get off the tube if nobody speaks to you and makes that connection’. (Participant 6, parent/carer)

‘I think, reminders that “If we do need to restrain you, it's not because you're choosing to do it” are helpful and “We know and understand you don't want this, but you have no choice, it doesn't mean that you need to kick and scream …” The illness often concludes that you need to be resisting as the only way of accepting [the feed] … And yeah, being reminded that you can sit there and accept it’. (Participant 16, PWLE)

‘I remember one patient, she had a really specific goal of getting back to her sport and we would work with her every feed and say “This is one step closer to getting back to this, we need to do this”, and um when she left the unit and went home she wrote to me and said how helpful that was on her darkest days’. (Participant 25, staff)

‘There needs to be more emphasis on, like, that they [staff] are against the illness, as it felt at times like they were against me and punishing me as a person. You have to split the person and the illness; we are not the same’. (Participant 16, PWLE)

### Importance of staff relationships with patients

The final theme identified was the importance of staff relationships with patients and how acts of human touch and simple kindness were able to reach the individual and not their illness.

‘Just physical contact. I know people sometimes are like “We can't hug patients”, but you can because if a patient is sobbing and they need comfort, their mum and dad aren't there. So that really helped me like being hugged and holding my hand’. (Participant 17, PWLE)

‘It's a really difficult one because the relationship with you, as a staff member, and the patient is really complex. You are there to help them but also doing this thing to them [restraint feeding] and you can see their distress and that can stay with you … So I would always check in and make sure they were ok and help them calm down, and that helped me to be ok too’. (Participant 25, staff)

Furthermore, these relationships appeared to be key to participants taking steps forward in their recovery, helping to foster trust and hope for recovery. These trusted staff were not always those assigned to the participants as their key workers, but rather staff members that the participants felt able to form a trusting relationship with.

‘The first one … my key worker was lovely, but my key nurse was, I mean she was nice, but she terrified me. So I wouldn't, I didn't want to speak to her … But for me there's one staff member … that I would like to talk to you about anything and if I was to make plan with her, I knew that I would stick to it because I didn't want to let her down’. (Participant 14, PWLE)

‘As a doctor, I always held hope for recovery otherwise what is the point of the job? Even with the sickest of patients. Um, I always held hope and they would look at you as if to say “Are you mad?” but then so many of them actually got better with the right support and that was so important to hold hope when others had lost it’. (Participant 33, staff)

‘When they finally agreed to change her key worker to one she liked, I was delighted and really felt that was a turning point in her recovery’. (Participant 2, parent/carer)

Conversely, the lack of trust in staff blocked progress in treatment, and coercion could alter a previously trusting relationship.

‘They just didn't get her at all. They didn't get the way she communicated and the way her brain worked. And just, you know, she didn't trust them and that is why she was stuck with restraints for so long’. (Participant 4, parent/carer)

‘Looking back, we had a close and compassionate relationship, but then she had to do my feeds, and everything changed, it's a different relationship when they have pinned you down and I remember thinking ‘Of all the people, you did that to me,’ and that is a tough one to come back from’. (Participant 18, PWLE)

‘I can remember we worked really hard to get the care plan right and manage some difficult changes and reduce the restraints. The first few days it worked and then it was the weekend and different staff. When I came back in on Monday she just said “They had no idea what they were doing”, and we were right back to square one’. (Participant 32, staff)

## Discussion

This is the first paper to qualitatively explore what good practice may look like when supporting patients to require NGT feeding no longer under restraint. The findings highlight three core themes, that of individualised care planning, good communication, and the importance of trust between the patient and the clinical team caring for them. These themes will now be explored further, with a view to shaping best practice recommendations.

### Individualised care planning

The use of evidence-based interventions (EBI) is at the core of day-to-day clinical practice for clinicians treating patients with eating disorders, and this aims to minimise harm and provide a high standard of care for the people we treat. For clinicians, there would ideally be multiple treatment options, with a hierarchy of success depending on various factors, that can be offered and discussed with each patient so treatment plans can be made in conjunction with the them. There is an evidence base for the treatment of eating disorders, but mostly psychotherapeutic for patients at higher weights and in out-patient settings.^14^ There are few models of in-patient care for eating disorders with a robust evidence base, leading to significant variation in practice. The exception is emerging evidence for enhanced cognitive behavioural therapy (CBT-E) for adolescents and adults with anorexia nervosa ^15^ and integrated enhanced cognitive behavioural therapy for adults with anorexia nervosa (I-CBTE).^16^ However, the risk remains with structured programmes that vital individualised aspects of a patient's treatment may be overlooked within these manualised programmes, and they have yet been adapted for patients with comorbid presentations.

For individuals with severe and complex eating disorder presentations who require admission to specialist mental health in-patient units, goals of admission are often based on measurable outcomes, such as weight restoration, reduction in specific behaviours or achieving a specific nutritional intake. Unfortunately, this may not always align with the person's goals and priorities at the time of treatment.^17^ This conflict in aims can lead to patients feeling that their treatment is not helpful and lead to conflict with healthcare professionals, entrenching resistance to treatment. Healthcare professionals in turn may feel they must continue restrictive practices to achieve weight restoration goals and avoid repeated weight loss whenever restrictive measures are rescinded. NGT feeding under physical restraint beyond the point of medical stabilisation is a good example of this reasoning. The participants in this study have highlighted that individualised care planning that has been done collaboratively with shared goals is what helped them to move on and no longer need this restrictive intervention. This aligns with the in-patient clinical guidance^18,19^ about the importance of care plans being developed collaboratively developed with the patient, and their family or carers if appropriate, and being individually tailored to meet their physical, psychological and social needs.

Conflict between the individual and their treating team can further be exacerbated by treatment programmes where patients, regardless of their presentation or personalities, are expected to follow the same treatment pathway as their peers. This demand for individualisation can present challenges for in-patient units where some coherence of approach and clarity around expectations can be helpful. However, when boundaries are challenged (such as consistently eating less than expected or needed for health) and patients resist the efforts of their treating healthcare professionals, restrictions can quickly escalate. The nature of in-patient care can increase the individual's sense of isolation and focus on their disorder. This is highlighted by a narrative account by O'Connell: ‘During these admissions especially, my inner world had become bleak and chaotic. My treatment reflected this, as it became ever more restrictive. The more I adopted the “anorexic role”, and the more time I spent under restrictive treatment conditions, the more I was distanced from “normal life”.’^20^ Indeed, research suggests that patients may resist treatment in order to try and exert some control over their situation^21^ or to assert a sense of autonomy,^22^ and this can lead to a cycle of resistance to treatment and more restrictive measures being put in place.^23^ Addressing the issue of control to break the deadlock so it does not escalate restriction may be helpful. This may include listening to people's preferences; allowing more choice in unrelated areas may also help increase dialogue even though some areas such as food intake may need to remain non-negotiable.

When restrictive practices or interventions are required, there are clear principles: ‘best interest of the patient’, ‘least restrictive practice’ and ‘proportionate to risk’ inherent in mental health legislation. Without informed consent, which is absent when a person is NGT-fed under physical restraint, clinicians utilise powers under mental health legislation. Nevertheless, for each use, they must still justify that they have reason to believe that their patient would benefit from this highly restrictive practice, that it is in their best interests and that a less restrictive option is unfeasible or ineffective, and then only use it as little as possible. Simply claiming that a programme of repeated NGT feeding under restraint is needed is unlikely to meet either ethical nor legal requirements of mental health legislation. Therefore, individualised care is needed to negotiate the optimal treatment and the least restrictive option at any given time.

### Good communication

Good communication and planning at the start of any mental health admission is important. Research suggests that the best practice is advance care planning between patients, carers and the clinical team where there is a joint discussion and agreement regarding and potential use of restrictive practices and the conditions under which they might occur.^6^ This should help individuals have a sense of ownership of the decision-making and understand that the clinicians are working with them from the start. This could become critical to success and collaboration if patients subsequently lose weight to the point of medical instability and clinicians need to consider overriding their wishes.^21,24,25^ Advance care planning can promote patient participation and prevent loss of agency when clinicians need to employ restrictive measures and help shift the narrative from ‘They are doing this to me, I didn't know this would happen, they just suddenly decided they would,’ to ‘This is my care plan, I did know this might happen and furthermore I do know why even if I can't agree.’^6^

It is important to acknowledge two key issues that may complicate the ability of a clinical team to communicate with their patients. First, severe malnutrition can exacerbate neurocognitive deficits such as rigid thinking and reduce the ability for patients to understand, retain and weigh up the information they are being given i.e. impair competence.^26,27^ This is not uncommon presentation in people with restrictive eating disorders such as anorexia nervosa. Second, a few patients will also have co-occurring psychiatric conditions such as autism spectrum condition (ASC) or depression, which may further influence how they respond to clinician expectations to increase intake.^12^

### Trust

The theme of trust was clearly identified within this research. There is little evidence in the scientific literature, which is because trust is difficult to quantify, unlike a person's weight or psychopathology scores. Some researchers have identified that trust is a ‘neglected concept’ in mental health services^28^ and that the betrayal of trust is identified as ‘normal part of care’ when working in mental health.^29^ However, patients and families all say that trust is critical to allowing individuals to do what their eating disorder is telling them they should not be allowing. Indeed, research highlighted that trust helps the development of good therapeutic relationships.^17,30^

### Strengths and limitations

This study adds to the literature examining what best practice may look like when NGT feeding under physical restraint is required. Qualitative interviews were used, and this allows for multiple perspectives, that of PWLE, parents/carers and clinicians, to be acknowledged in ways that other research methods cannot. Furthermore, research suggests that qualitative research can lead to a better understanding of what patients perceive as better mental health care.^24^

There are some limitations to this research. The study only represents the views of participants from England and may not be representative of views from other countries. There are relatively few participants, particularly in the PWLE group, because understandably recruitment was challenging as people were often reluctant to speak about traumatic and difficult experiences. The interview transcripts were generated by Microsoft Teams, and the audio recordings were used to corroborate the transcript; however, if there was a loss of connection, moments of the interview could have been missed. The PWLE were all diagnosed with anorexia nervosa, and the parents/carers were caring for someone with this diagnosis, and NGT feeding under physical restraint can also occur in patients with other mental health diagnoses; ^3^ therefore, this may not be an accurate reflection for all patients who require this intervention. One person reported receiving this intervention three decades ago, and treatments strategies for anorexia nervosa have developed and care philosophies have changed towards a recovery-oriented model ^31,32^ with less coercive practice.^23,33^ This participant's experience may not reflect modern practice; however, their participation is still valid as there may be people who historically experienced similar models of treatment and care who are still receiving care as a result. Finally, the themes arising from their interview were not markedly different from the themes from other PWLE who had more recent experiences of treatment.

One surprising aspect that was not reported by PWLE was the possible benefits of peer support, as research suggests that this can improve outcomes for those with mental illness^34^ and anorexia nervosa.^35^ This may be because it is not routinely employed in English in-patient mental health units. When surrounded by an equally unwell in-patient peer group and possibly also subject to NGT feeding under restraint, a sense of hopelessness may be fostered within an individual, and even evoke unhelpful competitiveness between patients for those requiring NGT feeding under restraint as a sign of loyalty and commitment to the eating disorder. Engaging with those who are in recovery and received this intervention but have then overcome their difficulties may foster hope and help patients to cope with or even avoid the intervention altogether.^35^

### Clinical implications – best practice

There were strong views regarding individualised care, the importance of good communication and trust. Treatment of an eating disorder requires both a patient's mental health concerns to be addressed and physical health restoration to take place. At times, clinicians will prioritise one over the other, i.e. if there is an immediate threat to life, physical health must be prioritised to prevent catastrophic outcomes. However, to help the group of people who experience NGT feeding under physical restraint, there should be a process of listening to their concerns, setting shared goals, individualising their care plans with advance discussion of this procedure and its place in treatment, negotiating at times where appropriate and problem solving at others, and giving as much choice as possible in other aspects of treatment. This process is therapeutic and patient-centred, as well as giving dignity to the individual. Delivering nutrition via NGT feeding under restraint is a highly aversive and restrictive process which, although lifesaving, runs a high risk of emotional distress for all, disempowerment, stripping patients of their human dignity, sense of autonomy and sense of self, inflicting trauma, and destroying relationships with clinical staff. It should therefore be employed with the greatest of sensitivity and care. The process of listening, working alongside our patients, giving choices wherever possible and developing shared goals is essential in the treatment of eating disorders in general; this is even more crucial in NGT feeding under restraint, rather than being antithetical to compulsion. It is also essential to prevent entrenchment of conflict between healthcare professionals and people undergoing NGT feeding under restraint, which may contribute to extended use of this coercive intervention.

### Future research

There is very little published regarding NGT feeding under physical restraint. By acknowledging the importance of individualised care, good communication and trust, the next steps would be to identify units where this is well established and to study how they do this. Researching these practical aspects in greater depth would be important to enable units to implement consistent and effective strategies to reduce the use of NGT feeding under restraint and improve the care they provide when it is needed, to minimise harm. Our study identifies some important mechanisms or approaches that could be built into clinical practice and be tested in clinical trials.

## Supporting information

Fuller et al. supplementary material Fuller et al. supplementary material

## Data Availability

The data that support the findings of this study are available from the corresponding author upon reasonable request.
