# Supplementary material for: The importance of individualised care, good communication and trust for reducing nasogastric tube feeding under physical restraint: qualitative multi-informant study
Source: BJPsych Open. 2024 Apr 17;10(3):e86. doi: 10.1192/bjo.2024.28 (PMC11060075; doi:10.1192/bjo.2024.28)
Supplement: Fuller et al. supplementary material [file S2056472424000280sup001.docx]

**Understanding compulsory naso-gastric nutrition**

Topic guide for expert by experience interviews

1. **Introductory questions** **(5-10 minutes)**

- *Example questions can include: what they’ve done today so far or how they are in general*
- *Specify that this is a safe space in which to talk openly about their experiences and recap on the importance of confidentiality, withdrawal and stopping if at any point they wish to have a break.*
- *Explain how the interviews are building on the survey data and will be exploring some areas in more detail*
- *Stress that nothing is too silly or weird to say; also that some questions may be silly or clumsy, we want their help to find a better way to ask*
- *This interview sees you an expert by experience and we hope that you can give unique insight into how practice can be improved.*
- *Ask if they have any questions before we begin*

1. **Understanding patient experience of being NGT fed under physical restraint (20 minutes)**

*First, we want to ask you what was your experience of being NGT fed under physical restraint?*

Tell me how it happened? – *note: this is opening question, I expect it will elicit a lot of answers to following questions, those should be asked if it doesn’t. But listen to the narrative first as it will orient you!*

- What did it feel like?
- What were you thinking at the time?
- Were you involved in the planning of your care and this intervention?
- Were you aware of the decision making process?
- Was anything done well?
- Was anything done badly or not so well?
- If you have had more than one experience, especially at different units, can you say how it was same or different experience? *Note: you might have to ask them to give narratives for a couple of different experiences*

1. **Impact of NG feeding under restraint (10-15 minutes)**

*What do you think can make NGT feeding under physical restraint a supportive intervention?*

- What advice would you give to make this experience less harmful at the time? If no response, prompt with: before, during and after, medications, staff support
- Any thoughts on what units can do that may help the feeding process more supportive or less bad?

- *What do you think are the potential harms of feeding under physical restraint?*
- Do you still think about it? Is talking about it distressing?
- Any thoughts on what may help other patients in this experience
- *How do you think being fed under restraint affects the staff on a unit?*
- *How do you think a patient being fed under restraint impacts parents / carers and other family members?*

1. **Patient characteristics and contextual factors (10 minutes)**

*You may have known about other people getting NG feeding under restraint, so this question is about people in general not just your own experience. What do you think some people require physical restraint for NGT feeding?*

- What would your observations be around who is likely to be fed under restraint? If no response, prompt with: what kinds of personality, what kinds of issues or attitudes,
- What are the patient circumstances that you feel might be relevant? If no response, prompt with: far from home/family/friends/transferred in from another unit on NG under restraint etc?
- Can factors on the unit make a difference? If no response, prompt with: peer group, staff dynamics, unit ethos, unit rigidity/rules. what sort of situation?
- Family
- Unit

1. **Reducing the need to NGT feed under physical restraint** **(20 minutes)**

*In your opinion, what helps patients to no longer require NGT feeding under physical restraint?*

- What do you think has helped you or other patients in the past? If no response, prompt with medication, family/friends reintegration, therapy approaches.

*In your opinion, what do you think can be done differently to prevent patients from needing NGT feeding under physical restraint?*

**Summing up**

And finally, is there anything else you’d like to discuss that we have not already talked about?

This is a very emotional topic; do you need further support around this? What support do you think you need? *Discuss what they need and how they’d get it.*

Thanks, and debrief (including options for support if necessary)

**Understanding compulsory naso-gastric nutrition**

Topic guide for parent/carer group interview

1. **Introductory questions** **(10 minutes)**

- *Allow the group to introduce themselves.*
- *Specify that this is a safe space in which to talk openly about their experiences and recap on the importance of confidentiality, withdrawal and stopping if at any point they wish to have a break.*
- *Explain how the interviews are building on the survey data and will be exploring some areas in more detail*
- *Show outline for session on a slide and explain that we hope to hear from everyone and apologise if there is a need to cut people short in order to get through the full interview as it is imperative that we get to the solutions*
- *Ask if they have any questions before we begin*

1. **Patient characteristics and contextual factors (20 minutes)**

*In your experience, why do some people require physical restraint for NGT feeding?*

- What are the patient characteristics that you feel are relevant?

If no response, prompt with: diagnosis / suspected diagnosis, nature of illness (AN), age/adolescent mind-set

- What are the patient circumstances that you feel are relevant?

   If no response, prompt with: far from home/family/friends?

- Can factors on the unit make a difference?

  If no response, prompt with: peer group, staff dynamics.

1. **NGT feeding under restraint** **(30 minutes)**

*We now want to explore how you think decisions to NGT feed under physical restraint come about?*

- Unit policy

If no response, prompt with: medical instability/malnutrition v’s refusal to eat/engage, hating the environment, being detained, should other factors be taken into consideration?

- *Do or should parents / carers get a say?*

If no response, prompt with collaboration with the team and communication from the team (were you aware of the decision making process?)

- What happens if you disagree with the MDTs decisions?

1. **Impact of NG feeding under restraint (20-30 minutes)**

*What do you think can make NGT feeding under physical restraint a supportive intervention?*

What measures can mitigate harms during the feeding process? If no response, prompt with: before, during and after, medications, staff support, family support

*What do you think are the potential harms of feeding under physical restraint?*

*How did this make you feel as a parent / carer, knowing your child was being fed in this way?*

*Do you still think about it? Is it distressing to talk about?*

*Would anything make it feel better?*

Or more reassuring?

1. **Reducing the need to NGT feed under physical restraint** **(20-30 minutes)**

*In your opinion, what helps patients to no longer require NGT feeding under physical restraint?*

What do you think has helped your child?

If no response, prompt with change of team, medication, family/friends reintegration, therapy approaches.

*In your opinion, what do you think can be done differently to prevent patients from needing NGT feeding under physical restraint?*

**Summing up**

Finally, is there anything else you’d like to discuss that we have not already talked about?

Thanks and debrief (including options for support if necessary)

**Understanding compulsory naso-gastric nutrition**

Topic guide for clinician interviews

1. **Introductory questions** *(helping people feel comfortable and at ease)* 5-10 minutes

- *Example questions can include: what they’ve done today so far or how they are in general*
- *Specify that this is a safe space in which to talk openly about their experiences and recap on the importance of confidentiality, withdrawal and stopping if at any point they wish to have a break.*
- *Explain how the interviews are building on the survey data and will be exploring some areas in more detail*
- *Ask if they have any questions before we begin*

1. **Understanding the ethos of the unit (10 minutes)**

*First, can I ask you how long you have worked here and what keeps you working here?*

- Can you tell me the qualities and values of the unit that you like? That you don’t like so much?
- How much do relationships with patients and families contribute to the culture or ethos of the unit?

What kind of policies/rules do your unit have about managing people who aren’t eating or drinking enough to maintain or gain weight?

- What happens in these cases?
- Are the rules flexible and individualised or apply to everyone?
- How is the event managed – in privacy? What do other patients know or witness? What preparations are there?
- What about the parents/carers?

1. **NGT feeding under restraint** **(10 minutes)**

*As you know, this research is about NG feeding where physical restraint is required. How do decisions to NGT feed a patient under physical restraint come about?*

- Who gets involved in the decision?
- Does everyone in the team get a say?
- Does the patient and family get a say?
- What happens if there is disagreement within the MDT?
- When would you seek independent advice?

1. **Impact of NG feeding under restraint (15 minutes)**

*What do you think can make NGT feeding under physical restraint a more supportive intervention?*

*What do you think are the potential harms of feeding under physical restraint?*

- *Prompt: psychological, traumatisation, physical, relational, entrenching conflict*
- What measures can mitigate harms during the feeding process? If no response, prompt with: before, during and after, medications, staff support, reduced frequency
- Any thoughts on what else may help patients?

- What are your thoughts about the impact of feeding under restraint on staff
- *What do you do to mitigate the harms for staff? Which staff are the most effected? E.g. people doing restraint, staff member passing NGT, dietitian prescribing the feed, etc…Any thoughts on what else may help staff?*

What are your thoughts about the impact of feeding under restraint on parents and carers?

1. **Patient characteristics and contextual factors (10 minutes)**

*In your experience, why do some people require physical restraint for NGT feeding?*

- What are your observations about the patients who end up being NGT fed under physical restraint?  If no response, prompt with: diagnosis / suspected diagnosis, nature of illness (AN), age/adolescent mind-set
- What are the patient circumstances that you feel are relevant? If no response, prompt with: far from home/family/friends?
- Which factors on the unit make a difference? If no response, prompt with: dynamics, conflict, familiarity of patient’s responses, relationship with patients, peer group, staff dynamics.

1. **Improving NGT practice (10 mins)**

*In your opinion, what helps patients to no longer require NGT feeding under physical restraint?*

- What do you think has helped patients in the past? If no response, prompt with medication, family/friend’s reintegration, therapy approaches.

*In your opinion, what do you think can be done differently to prevent patients from needing NGT feeding under physical restraint?*

*Is there anything else you would like to say about what can be done differently?, to help NGT feeding be less frequently used or less traumatic?*

**Summing up**

- Is there anything else you’d like to discuss that we have not already talked about?
- You might feel emotional after this, how will you get support?
- Thanks and debrief (including options for support if necessary)
